# Prevalence of Risk of Iron Deficiency

**DOI:** 10.1080/24740527.2026.2696247

**Published:** 2026-07-20

**Authors:** Bashar Alobeid, Ruhaim Ali, Norman Buckley

**Affiliations:** a Department of Health Sciences, McMaster University, Hamilton, Canada; b Department of Anesthesia, Michael G. DeGroote Institute for Pain Research and Care, McMaster University, Hamilton, Canada

**Keywords:** Iron deficiency, chronic pain, ferritin, serotonin, women’s health, sex and gender, fibromyalgia, pain management

## Abstract

**Background:**

Iron deficiency (ID), a common cause of anemia, disproportionately affects women of reproductive age and is characterized by symptoms such as fatigue, cognitive fog, migraines, restless‑leg syndrome, and musculoskeletal pain. Some of these are also common to chronic pain syndromes. Iron is essential for the synthesis of multiple neurotransmitters, suggesting a mechanistic link between ID and pain perception.

**Methods:**

This single‑center cross-sectional study assessed the prevalence of risk factors for iron deficiency among 81 women attending the Michael G. DeGroote Pain Clinic. Participants completed a validated questionnaire adapted from Dr. Toby Richards’ Iron Clinic, which evaluated risk factors including heavy menstrual bleeding, prior iron‑deficiency status, and supplementation history. Patients were defined as being at high risk for ID if they endorsed two or more positive symptoms within Question 14 (menstrual‑bleeding characteristics).

**Results:**

Overall, 54% of participants were identified as high risk for iron deficiency. 46% reported having restless‑leg syndrome, 47% reported a history of anemia or ID, 41% had taken oral iron supplements, and 15% were receiving iron‑infusion therapy. These results indicate that a substantial proportion of women with chronic pain may also have unrecognized ID.

**Discussion:**

Since iron’s role in neurotransmitter metabolism provides a plausible biological mechanism for some symptomatology, and there is overlap of the symptoms of ID and chronic pain conditions, routine screening for ID, along with patient education, dietary optimization, oral iron supplementation, and intravenous iron therapy, should be considered as part of integrative pain‑management strategies. Cost-effectiveness analyses support ferritin screening as a feasible approach in this population, highlighting the potential for improved patient outcomes.

## RÉSUMÉ

**Contexte :** La carence en fer, cause fréquente d’anémie, touche de manière disproportionnée les femmes en âge de procréer et se manifeste notamment par des symptômes tels que de la fatigue, un brouillard cognitif, des migraines, un syndrome des jambes sans repos et des douleurs musculosquelettiques. Certains de ces symptômes sont également présents dans les syndromes de douleur chronique. Le fer est essentiel à la synthèse de multiples neurotransmetteurs, ce qui donne à penser qu’il existe un lien mécanistique entre la carence en fer et la perception de la douleur.

**Méthodes :** Cette étude transversale monocentrique a évalué la prévalence des facteurs de risque de carence en fer chez 81 femmes suivies à la Michael G. DeGroote Pain Clinic. Les participantes ont rempli un questionnaire validé, adapté de celui utilisé à l’Iron Clinic du Dr Toby Richards, qui évaluait notamment les facteurs de risque suivants : saignements menstruels abondants, antécédents de carence en fer et antécédents de supplémentation en fer. Les patientes étaient considérées comme présentant un risque élevé de carence en fer si elles avaient signalé au moins deux des caractéristiques énumérées à la question 14, qui portait sur les saignements menstruels.

**Résultats :** Dans l’ensemble, 54 % des participantes ont été considérées comme présentant un risque élevé de carence en fer. Parmi ailleurs, 46 % ont déclaré présenter un syndrome des jambes sans repos, 47 % ont signalé des antécédents d’anémie ou de carence en fer, 41 % avaient déjà pris des suppléments de fer par voie orale et 15 % recevaient un traitement par perfusion de fer. Ces résultats indiquent qu’une proportion importante de femmes souffrant de douleur chronique pourrait également présenter une carence en fer non reconnue.

**Discussion :** Comme le rôle du fer dans le métabolisme des neurotransmetteurs constitue un mécanisme biologique plausible pouvant expliquer certains symptômes, et que les symptômes de la carence en fer et ceux de la douleur chronique se chevauchent, le dépistage systématique de la carence en fer, de même que l’éducation des patientes, l’optimisation de l’alimentation, la supplémentation en fer par voie orale et l’administration de fer par voie intraveineuse, devraient être envisagés dans le cadre de stratégies intégrées de prise en charge de la douleur. Les analyses coût-efficacité indiquent que le dépistage fondé sur la mesure de la ferritine constitue une approche réalisable dans cette population et pourrait contribuer à améliorer les résultats cliniques pour les patientes.

## Introduction

In 2021, a Lancet study found that 1.92 billion people globally were suffering from anemia with a disproportionate impact on women. Iron deficiency made up for 66% of these cases, with a gender breakdown of 825 million women and 444 million men. The differing burden is attributed to gynecological disorders and maternal hemorrhage as significant contributors to the extensive anemia burden for women of reproductive age.^1^ The clinical entity of non-anemic iron deficiency can also be associated with a variety of symptoms and is more common in women than men.^2^

Women have a greater incidence of chronic pain conditions than men.^3^ A recent review comments on a variety of possible reasons for this. One possible mechanism is the role of iron in neurotransmitter synthesis. Serotonin for example is important for mood as well as pain sensation.^4^ Serotonin is a crucial neurotransmitter involved in regulating mood, pain perception, gastrointestinal function, and overall psychological well-being. Notably, tryptophan hydroxylase (TrPH) requires ferrous iron (Fe^2+^) as a cofactor to function effectively. Alterations in iron levels can significantly affect TrPH activity, impairing serotonin synthesis. Once synthesized, serotonin exerts its effects through a wide range of receptor subtypes distributed across both the central and peripheral nervous systems.^5^

Iron is also a key factor in the production of a number of other neurotransmitters including dopamine, norepinephrine, and epinephrine, as well as the function of signaling pathways. Iron is essential in synthesizing dopamine, serving a core function in catalyzing the production of the precursor amino acid L-DOPA. Meanwhile, dopamine itself serves as a norepinephrine precursor, thus showcasing iron’s important role within the nervous system. As a result of iron’s integral role within the nervous system, it becomes important that more light is shed on how iron deficiency may influence neurological and neurochemical processes.^6^ The relationship between iron status and nervous system outcomes remains very complex. A comprehensive discussion is beyond the scope of this study.

The overlap of symptoms between iron deficiency and chronic pain syndromes further suggests a potential mechanistic link. Some chronic pain syndromes are more common in women.^7^ Iron deficiency has been associated with symptoms many of which are also characteristic of chronic pain conditions, such as weakness, fatigue, reduced exercise performance, difficulty in concentrating, poor work productivity, neurocognitive dysfunction including irritability, fibromyalgia syndrome, restless‑leg syndrome, symptom persistence in patients treated for hypothyroidism, poor neurodevelopmental outcomes in infants born to mothers with iron deficiency and migraines.^8,9^ A study by Yao et al. showcased that iron‑deficiency anemia is associated with fibromyalgia occurrence by an adjusted hazard ratio of 1.18, with anemia alone not completely accounting for this association.^10^ In addition, a history of iron deficiency is a statistical predictor that is significantly associated with symptoms such as migraine, headache, recurrent abdominal pain, dysmenorrhea, and diverse chronic pain conditions.^11,12^

Given its association with these symptoms, the recognition of the several biological mechanisms by which an effect may occur, and the treatability of the condition, addressing iron deficiency may provide a new path toward improving pain management. Iron status remains an underrecognized factor in chronic pain assessment and management. Women are at increased risk of iron deficiency and are more at risk of experiencing chronic pain syndromes. Dr. Toby Richards has been exploring this particularly with female athletes and has developed questionnaires to screen for the risk of ID.^13^

We report a study that investigated the prevalence of risk of iron deficiency among women experiencing chronic pain and attending the Michael G. DeGroote Pain Clinic.

## Materials and methods

### Study design and consultation

This study was conducted as a cross-sectional analysis to assess the frequency of risk of iron deficiency in women with chronic pain. Dr. Richards’ team provided a comprehensive questionnaire to identify individuals at risk of iron deficiency. This questionnaire was refined with a focus on the identification of iron deficiency in female patients attending the Pain Clinic. The questionnaire determines high risk for iron deficiency through known risk factors: heavy menstrual bleeding, previous iron status, and iron supplementation. Previous studies identified heavy menstrual bleeding as a significant predictor of anemia and progression into ID.^14^ Patients were asked to report any of the four major symptoms (need double sanitary protection, need to frequently change your protection, had or worried about flooding through to clothes or bedding, pass large blood clots). Answering yes to 2 or more items was considered to describe heavy menstrual bleeding and therefore high risk for experiencing iron deficiency.

### Ethical considerations

All researchers completed Good Clinical Practice (GCP) and ethics training. The study was reviewed and approved by the Hamilton Integrated Research Ethics Board (HiREB certificate number #18098).

### Methods

The sample size for the cross-sectional study was determined through a combination of statistical calculations and feasibility assessments. The target sample was chosen to ensure that the results would allow for meaningful conclusions regarding the prevalence of women with chronic pain at high risk for iron deficiency at the Pain Clinic. The study methodology, including data collection methods, was finalized after consultations with Dr. Behnam Sadeghirad. The following formula was used for sample size calculation:

(1) $$n=\frac{{Z^{2}}_{\left(1-\frac{\alpha }{2}\right)P\left(1-P\right)}}{d^{2}}$$

where α represents the confidence interval of 95%, *Z* represents the *Z*-score of the desired confidence interval (0.95), *P* represents the assumed proportion/prevalence of women with iron deficiency (0.3), and *d* represents the margin of error or precision (10%). A sample size of 81 was chosen.

Patient recruitment was carried out through convenience sampling. Investigators attended the clinic on days when the clinic staff advised that significant numbers of patients matching the eligibility criteria were scheduled to attend. Eligible participants included biological women between the ages of 18–49 years old attending the Michael G. DeGroote Pain Clinic for a chronic pain treatment, intervention, information session, or consultation. Within this group, there were no further exclusion criteria. Potential participants were informed by clinic staff of the ongoing study. The investigators then obtained written consent from each participant and administered the survey. Participants were approached as they arrived with no overt selection process.

## Results

**Figure 1.**
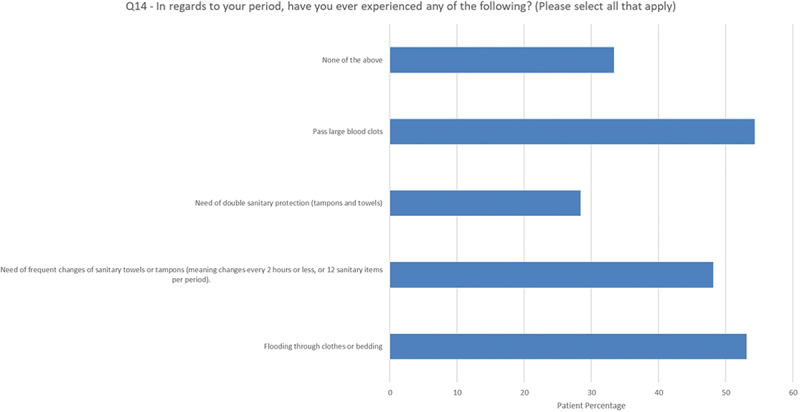
Self-reported menstrual bleeding characteristics among recruited participants. Bar chart showing the percentage of participants reporting symptoms associated with heavy menstrual bleeding. Participants could select more than one response (if applicable); therefore, percentages do not total 100%.

81 patients were recruited into the study. 47% (38) reported a history of anemia or iron deficiency in the last 2 years, 41% (33) reported having taken oral iron supplementation, and 15% (12) had undergone iron infusion therapy in the last 2 years. 17% (14) of the patients were following a vegetarian, vegan, or pescatarian diet. 46% (37) reported having restless legs as a symptom. 54% (44) of our patients answered yes to 2 of our 4 major menstrual symptoms associated with iron deficiency, with the distribution outlined in Figure 1.

**Figure 2.**
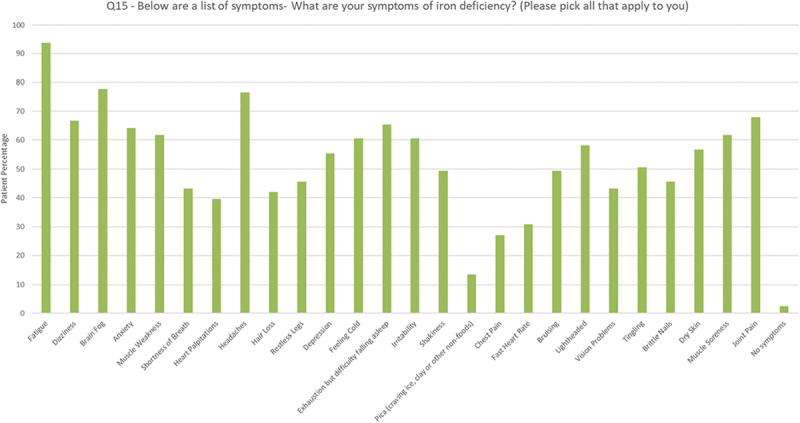
Self-reported symptoms of iron deficiency among recruited participants. Bar chart showing the percentage of participants reporting individual symptoms of iron deficiency. Participants could select more than one symptom; therefore, percentages do not total 100%.

Additionally, many of our patients reported suffering from symptoms associated with iron deficiency. The distribution of these symptoms is outlined in Figure 2.

## Discussion

This study’s main finding, that 54% of surveyed female chronic pain patients were deemed at high risk for iron deficiency, sheds light on the prevalence of this issue. Considering the social determinants of health, there may be systemic issues placing certain populations at higher risk of iron deficiency. *Barton et al*. have highlighted this issue by showcasing how the prevalence of NAID among women varied significantly when accounting for demographic criteria such as ethnic and financial background, especially among Black and Hispanic demographics.^2^ Additionally, Wen et al. conducted a retrospective cohort study in laboratory screening for non-pregnant females in Ontario, Canada, that found low household income to be associated with the greatest odds of anemia and the lowest likelihood of being screened.^14^ This makes it quite clear that iron deficiency is more than simply a biomedical issue, but rather a condition disproportionately affecting people of different backgrounds. Exploring potential confounding factors involves understanding dietary sources of iron. A 2016 review by *Pawlak et al*. found that vegetarians had a higher prevalence of depleted iron stores and iron‑deficiency anemia, which was especially true for premenopausal vegetarian women.^15^ However, well-planned plant-based diets may still be nutritionally adequate and a vegetarian or vegan diet may not be a clear-cut risk factor for ID.^16^ In pregnancy, iron requirements rise dramatically as blood volume grows while the offspring grows, increasing the risk of maternal ID.^17^ In North America, the estimated prevalence of ID during pregnancy is believed to be greater than 50%, with almost 12% of pregnancies being impacted by iron‑deficiency anemia.^18^ Maternal ID is associated with adverse pregnancy outcomes, with the developing fetus’ brain at special risk of ID-related complications, such as slower processing speed and poorer bonding.^17^

Social determinants of health such as low socioeconomic status, food insecurity, limited access to healthcare, and stigmatized menstrual health play a role in how iron deficiency manifests in a person, as well as how they go about addressing their condition. Many of our patients affirmed this, with a recurring concern being the limited awareness about the condition. Routine checkups may not include testing ferritin levels, and many symptoms associated with iron deficiency are often nonspecific or shared with other mineral deficiencies or conditions which makes it hard to identify iron deficiency as a potential problem.^19^ ID may also coexist with other deficiencies, such as vitamin B12. One study found low serum B12 and ferritin levels associated with disease severity and neuropathic pain in fibromyalgia patients.^20^ Patients with chronically painful conditions such as spondyloarthritis often carry the comorbidity of anemia which has also been explored with some research finding that the use of non-steroidal anti-inflammatory drugs offers the potential for blood loss and iron depletion.^21^

Current treatment options for addressing iron deficiency are sometimes problematic. Up to 60% of patients complain about symptoms associated with oral iron supplementation such as nausea and constipation which can affect compliance with therapy, with 50% of patients abandoning oral supplementation.^22^ Iron infusion therapy attempts to rectify this through a single-session appointment that provides iron intravenously and effectively, but this may not be readily available. Both treatment options provide iron replacement, but do not address the root causes that led to iron deficiency. These include deficiencies in dietary intake resulting from either food insecurity or ingesting a primarily vegetarian/vegan-based diet. This combined with a general lack of emphasis placed on menstrual health results in many of the patients redeveloping iron deficiency issues even after having been treated.^23^

Recent initiatives launched by the Ontario government such as “Raise the Bar” have attempted to rectify this lack of awareness surrounding iron deficiency. Launched in September 2024, it establishes a plan to standardize routine care as well as provide increased education to healthcare workers regarding iron deficiency. This has included establishing higher clinical decision limits for ferritin testing, with levels below 30 µg/L now signifying iron deficiency. Furthermore, an updated interpretive guidance list regarding other ferritin levels has also been released to help give doctors and patients a better understanding of the likelihood and potential severity of iron deficiency in patients with ferritin levels between the 30–100 µg/L range.^24^ Noteworthy as well are the resources and educational programs provided to various members of the healthcare system. The online platform *Hemequity* was launched to help equip physicians with the appropriate knowledge regarding the diagnosis and treatment of iron deficiency.

These initiatives are aimed at addressing the issues associated with the presentation of iron deficiency among women of reproductive age. However, more work still needs to be done to address iron deficiency, especially among women with chronic pain. A retrospective study published in 2024 by the American Journal of Hematology analyzed the prevalence of iron deficiency in non-pregnant Ontario women by measuring their ferritin levels. At ferritin levels in the guidelines set by the “Raise the Bar” initiative, 38.3% of all Ontario women were reported to experience NAID.^14^ Our observation that 54% are at risk of ID seems consistent with this, given that actual ferritin levels may differ, and we are also examining a symptomatic population rather than a general population.

A recently published cross-sectional study explored the relationship between ID and chronic pain patients, finding a prevalence of 58.8% in a population of 82 patients attending a chronic pain outpatient appointment using venous blood samples. However, no statistically significant association between ID status and fatigue or quality‑of‑life measures used within the study was found.^25^ It is interesting to note that the prevalence of ID in this study with a similar patient population and size is comparable to our observed prevalence of risk of ID of 54% across 81 chronic pain patients.

There have been several recent articles identifying connections between iron deficiency and a variety of pain conditions that are often more common in women. The survey we report has shown that a simple set of screening questions can identify patients who are potentially at risk of iron deficiency and should be investigated further. What is not yet known is whether correction of this deficiency will lead to resolution of these symptoms or if other interventions may be required, given that chronic pain is often a complex condition. In fact, while this biological mechanism is potentially readily treatable, given our understanding of pain as a complex bio-psycho-social phenomenon, if this is reversed at a late stage, it may still be necessary to enter into treatment pathways with a focus on recovery of function.

It is our belief that routine screening for iron deficiency in women living with chronic pain should be implemented. Interestingly, some investigators have run computer models with regard to ferritin screening and believe that it is cost-effective in terms of quality-adjusted life years, although this has yet to be borne out of clinical studies.^26^ Having identified that more than half of surveyed women may be at risk of iron deficiency; we are now engaged in discussions with clinic personnel to include biomarker screening in regular day‑to‑day practice.

### Strengths

This screening was well accepted by patients and is simple and easy to interpret.

### Limitations

This is a single-site study. While attempts were made to ensure that all eligible patients were approached, this may not have always occurred. Risk for iron deficiency is not the same as iron deficiency, so next steps require ferritin levels. Furthermore, patients were not directly assessed for their chronic pain conditions, given that they were all attending the pain clinic and therefore the presence of pain was taken as a given. This limited the possibility of building further connections between chronic pain severity, iron deficiency rates, and specific conditions. It has yet to be demonstrated that iron replacement to a criterion will modify the pain experience of these patients.

## Conclusion

Given the role of iron in the production of multiple neurotransmitters, all of which are associated with a variety of symptoms to pain conditions, it is quite possible that iron deficiency plays a role in the experience of chronic pain. Furthermore, we also suggest that preventative options such as improved dietary intervention, routine screening, and education should be integrated early on into standard care pathways to identify potentially correctable contributors to chronic pain. Findings from this work may support future integrative pain management strategies that include definitive screening for and correction of iron deficiency as part of a broader biopsychosocial approach. This needs to be tested in high-quality studies.

## Supplementary Material

Qualtrics BRID Survey.pdf

Qualtrics BRID Survey Participant Answers.pdf

## References

[1] GBD 2021 Anaemia Collaborators. Prevalence, years lived with disability, and trends in anaemia burden by severity and cause, 1990–2021: findings from the Global Burden of Disease Study 2021. Lancet Haematol. 2023;10(9):e713–8. doi: 10.1016/S2352-3026(23)00160-6. 37536353 PMC10465717

[2] Barton JC, Wiener HH, Acton RT, Adams PC, Eckfeldt JH, Gordeuk VR, Harris EL, McLaren CE, Harrison H, McLaren GD, et al. Prevalence of iron deficiency in 62,685 women of seven race/ethnicity groups: the HEIRS study. PLOS ONE. 2020;15(4):e0232125. Published 2020 Apr 23. 32324809 10.1371/journal.pone.0232125 PMC7179917

[3] Gender Differences in Chronic Pain Conditions. International Association for the Study of Pain (IASP). 2024. https://www.iasp-pain.org/resources/fact-sheets/gender-differences-in-chronic-pain-conditions/. Published 2024 June 28.

[4] Stieger A, Asadauskas A, Luedi MM, Andereggen L. Women’s pain management across the lifespan-a narrative review of hormonal, physiological, and psychosocial perspectives. J Clin Med. 2025;14(10):3427. Published 2025 May 14. 40429422 10.3390/jcm14103427 PMC12112123

[5] Gonçalves S, Nunes-Costa D, Cardoso SM, Empadinhas N, Marugg JD. Enzyme promiscuity in serotonin biosynthesis, from bacteria to plants and humans. Front Microbiol. 2022;13:873555. Published 2022 Apr 14. 35495641 10.3389/fmicb.2022.873555 PMC9048412

[6] Berthou C, Iliou JP, Barba D. Iron, neuro-bioavailability and depression. EJHaem. 2021;3(1):263–275. Published 2021 Dec 5. doi: 10.1002/jha2.321. 35846210 PMC9175715

[7] Ruschak I, Montesó-curto P, Rosselló L, Aguilar Martín C, Sánchez-Montesó L, Toussaint L. Fibromyalgia syndrome pain in men and women: a scoping review. Healthcare (Basel). 2023;11(2):223. Published 2023 Jan 11. 36673591 10.3390/healthcare11020223 PMC9859454

[8] Balendran S, Forsyth C. Non-anaemic iron deficiency. Aust Prescr. 2021;44(6):193–196. doi: 10.18773/austprescr.2021.052. 35002031 PMC8671013

[9] Al-Qassab ZM, Ahmed O, Kannan V, Ullah N, Geddada S, Ibrahiam AT, Nwosu M. Iron deficiency anemia and migraine: a literature review of the prevalence, pathophysiology, and therapeutic potential. Cureus. 2024;16(9):e69652. doi: 10.7759/cureus.69652. Published 2024 Sep 18. 39429346 PMC11488462

[10] Yao WC, Chen HJ, Leong KH, Chang KL, Wang YTT, Wu LC, Tung PY, Kuo CF, Lin CC, Tsai SY. The risk of fibromyalgia in patients with iron deficiency anemia: a nationwide population-based cohort study. Sci Rep. 2021;11(1):10496. doi: 10.1038/s41598-021-89842-9. PMC8131369 34006944

[11] Champion D, Bui M, Aouad P, Teng A, Walters A, Karroum E, Tan A, Yang Z, Joyce E, Jaaniste T, et al. Associations between lifetime histories of iron deficiency, anxiety, depression and multiple pain conditions: an observational study using a large-scale national database. J Pain Res. 2025;18:3781–92. Published 2025 Jul 30. doi: 10.2147/JPR.S497122. 40756431 PMC12318526

[12] Champion GD, Jaaniste T, Tan AC. Pain in women: relevance of history of iron deficiency: comment on Laughey et al. (2025). Pain Rep. 2025;11(1):e1383. Published 2025 Dec 23. 41450706 10.1097/PR9.0000000000001383 PMC12737852

[13] Munro MG, Mast AE, Powers JM, Kouides PA, O’Brien SH, Richards T, Lavin M, Levy BS. The relationship between heavy menstrual bleeding, iron deficiency, and iron deficiency anemia. Am J Obstet Gynecol. 2023;229(1):1–9. doi: 10.1016/j.ajog.2023.01.017. 36706856

[14] Wen S, Nisenbaum R, Weyand AC, Tang GH, Auerbach M, Sholzberg M. High prevalence of iron deficiency and socioeconomic disparities in laboratory screening of non-pregnant females of reproductive age: a retrospective cohort study. Am J Hematol. 2024;99(8):1492–1499. doi: 10.1002/ajh.27352. 38695834

[15] Pawlak R, Berger J, Hines I. Iron status of vegetarian adults: a review of literature. Am J Lifestyle Med. 2016;12(6):486–498. Published 2016 Dec 16. doi: 10.1177/1559827616682933. 30783404 PMC6367879

[16] López-Moreno M, Castillo-García A, Roldán-Ruiz A, Viña I, Bertotti G. Plant-based diet and risk of iron-deficiency anemia. A review of the current evidence and implications for preventive strategies. Curr Nutr Rep. 2025;14(1):81. Published 2025 Jun 18. 40528105 10.1007/s13668-025-00671-y PMC12174276

[17] Georgieff MK. Iron deficiency in pregnancy. Am J Obstet Gynecol. 2020;223(4):516–524. doi: 10.1016/j.ajog.2020.03.006. 32184147 PMC7492370

[18] Benson AE, Lo JO, Caughey AB. Iron deficiency and iron deficiency anemia during pregnancy-opportunities to optimize perinatal health and health equity. JAMA Netw Open. 2024;7(8):e2429151. Published 2024 Aug 1. 39163051 10.1001/jamanetworkopen.2024.29151 PMC11380762

[19] Tang GH, Sholzberg M. Iron deficiency anemia among women: an issue of health equity. Blood Rev. 2024;64:101159. 38042684 10.1016/j.blre.2023.101159

[20] Bingol MK, Akturk S, Buyukavci R, Zontul S. The relationship of serum vitamin B12 and ferritin levels with disease severity and neuropathic pain in fibromyalgia syndrome. J Coll Physicians Surg Pak. 2025;35(2):209–212. doi: 10.29271/jcpsp.2025.02.209. 39936200

[21] Safarova KN, Dorogoykina KD, Rebrov AP. Is anemia a clinical marker of NSAIDs-induced upper gastrointestinal lesions in patients with spondyloarthritis? Almanac Clin Med. 2019;47(5):410–418. doi: 10.18786/2072-0505-2019-47-037.

[22] Bloor SR, Schutte R, Hobson AR. Oral iron supplementation—gastrointestinal side effects and the impact on the gut microbiota. Microbiol Res. 2021;12(2):491–502. doi: 10.3390/microbiolres12020033.

[23] Kumar SB, Arnipalli SR, Mehta P, Carrau S, Ziouzenkova O. Iron deficiency anemia: efficacy and limitations of nutritional and comprehensive mitigation strategies. Nutrients. 2022;14(14):2976. Published 2022 Jul 20. 35889932 10.3390/nu14142976 PMC9315959

[24] Tang GH, Phillips Z, Poutanen S, Yip P, Brinc D, Goh E, Cheung M, Pai M, Oleschuk C, Beriault D, et al. ‘Raise the Bar,’ a Canadian intervention to combat disparities in the recognition and management of iron deficiency: development of the intervention, feasibility and implementation. Br J Haematol. 2025;207(5):2219–2223. doi: 10.1111/bjh.70150. 40936490 PMC12624194

[25] Wyssusek KH, Woods CA, Minard ET, Lee J, Pelecanos A, Gray P. A cross-sectional study of the relationship between iron deficiency anaemia and chronic pain. Anaesth Intensive Care. 2024;52(6):369–376. doi: 10.1177/0310057X241263612. 39233562

[26] Wang D, Sra M, Glaeser-Khan S, Wang DY, Moshashaian-asl R, Ito S, Cuker A, Goshua G. Cost-effectiveness of ferritin screening thresholds for iron deficiency in reproductive-age women. Am J Hematol. 2025;100(7):1132–1140. doi: 10.1002/ajh.27686. 40235279 PMC12146817

