# Supplementary material for: Prevalence of Risk of Iron Deficiency
Source: Can J Pain. 2026 Jul 20;10(1):2696247. doi: 10.1080/24740527.2026.2696247 (PMC13387105; doi:10.1080/24740527.2026.2696247)
Supplement: Qualtrics BRID Survey.pdf [file UCJP_A_2696247_SM6309.pdf]

Participant Information and Consent Form

Are you a woman aged 18 - 49 years old?

Yes
No

Menstruation

To your knowledge, have you ever had anaemia or iron deficiency in the past 2 years?

Yes
No

Have you taken oral iron tablets in the past 2 years?

Yes
No

Have you ever had an iron infusion?

Yes
No

How many years ago was your iron infusion?

Block 7

Approximately how many periods have you had in the last 12 months?

	0	1	2	3	4	5	6	7	8	9	10	11	12	13	14	15	16
Number of periods																	

In regards to your period, have you ever experienced any of the following? (Please select all that apply)

Flooding through clothes or bedding
Need of frequent changes of sanitary towels or tampons (meaning changes every 2 hours or less, or 12 sanitary items per period).

Need of double sanitary protection (tampons and towels)
Pass large blood clots
None of the above

Iron Deficiency Symptoms and Impact

Below are a list of symptoms- What are your symptoms of iron deficiency? (Please pick all that apply to you)

Fatigue
Dizziness
Brain Fog
Anxiety
Muscle Weakness
Shortness of Breath
Heart Palpitations
Headaches
Hair Loss
Restless Legs
Depression
Feeling Cold
Exhaustion but difficulty falling asleep
Irritability
Shakiness
Pica (craving ice, clay or other non-foods)
Chest Pain
Fast Heart Rate
Bruising
Lightheaded
Vision Problems
Tingling
Brittle Nails
Dry Skin
Muscle Soreness
Joint Pain
No symptoms

Block 6

Have you donated blood in the past 2 years?

Yes
No

Do you follow a vegetarian, vegan, pescatarian or similar diet?

Yes
No

Block 5

Have you ever had a pregnancy?

Yes
No

How many children do you have?

	0	1	2	3	4	5	6	7	8	9	10
Number of children											

How many years ago was your last child born?

At your last childbirth, were you known to be iron deficient or anaemic?

Yes
No

At your last childbirth, did you suffer from hair loss?

Yes
No

At your last childbirth, did you suffer from postpartum depression?

Yes
No

Results

Would you like to see your results from the questionnaire?

Yes
No

According to the questionnaire, you have been identified as at high risk of experiencing iron deficiency. Please remember that this is a simple screening tool on risk, and in no way is meant to confirm the presence or absence of iron deficiency. We would recommend discussing these results with your family doctor.

According to the questionnaire, you have not been identified as at high risk of experiencing iron deficiency. Please remember that this is a simple screening tool on risk, and in no way

is meant to confirm the presence or absence of iron deficiency.

Powered by Qualtrics
