# Supplementary material for: Prevalence of Risk of Iron Deficiency
Source: Can J Pain. 2026 Jul 20;10(1):2696247. doi: 10.1080/24740527.2026.2696247 (PMC13387105; doi:10.1080/24740527.2026.2696247)
Supplement: Qualtrics BRID Survey Participant Answers.pdf [file UCJP_A_2696247_SM6308.pdf]

BRID Questionnaire / Page 1

Responses: 81

Are you a woman aged 18 - 49 years old? 81

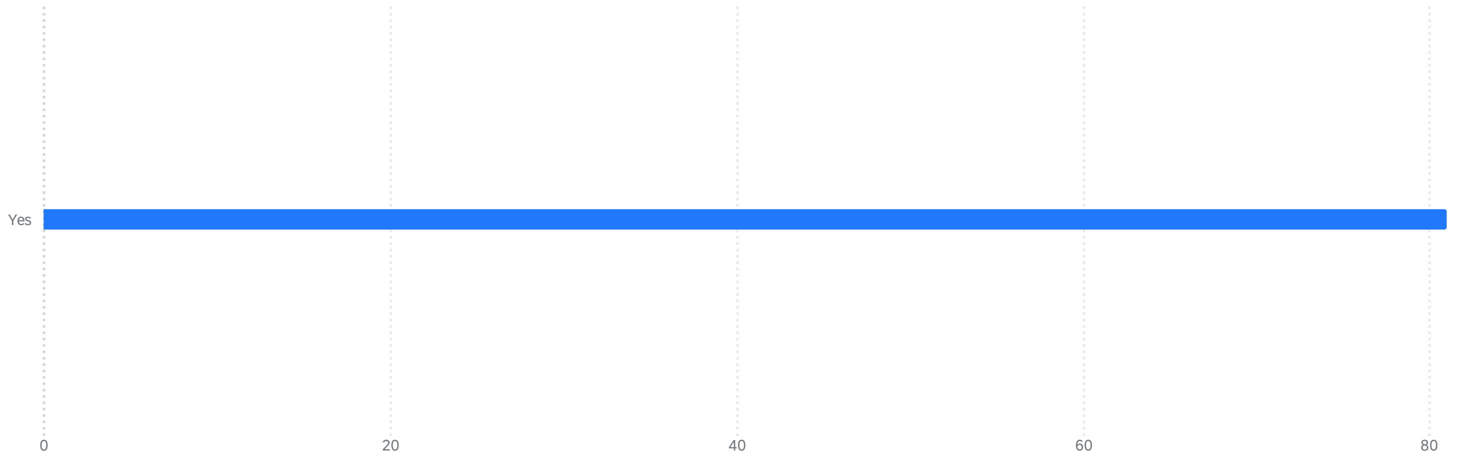

Are you a woman aged 18 - 49 years old? 81

Q40 - Are you a woman aged 18 - 49 years old?	Percentage	Count
Yes	100%	81

Are you a woman aged 18 - 49 years old? 81

Are you a woman aged 18 - 49 years old?	Average	Minimum	Maximum	Count
Yes	1.00	1.00	1.00	81

I consent to participate in this research project

This widget is not configured correctly. Please check your widget configuration.

I consent to participate in this research project ⓘ ⚠

This widget is not configured correctly. Please check your widget configuration.

I consent to participate in this research project ⓘ ⚠

This widget is not configured correctly. Please check your widget configuration.

Please sign here ⓘ ⚠

This widget is not configured correctly. Please check your widget configuration.

First name

This widget is not configured correctly. Please check your widget configuration.

Last name

This widget is not configured correctly. Please check your widget configuration.

Email address (for the purpose of communicating the results)

This widget is not configured correctly. Please check your widget configuration.

Date of birth (dd/mm/yyyy)

This widget is not configured correctly. Please check your widget configuration.

To your knowledge, have you ever had anaemia or iron deficiency in the past 2 years? 81

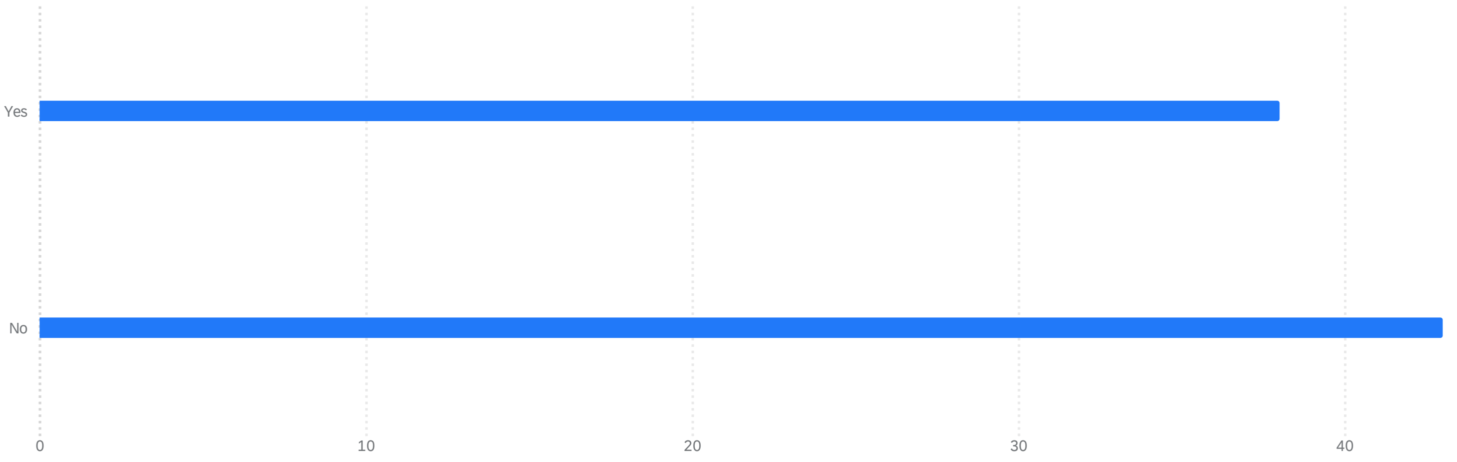

To your knowledge, have you ever had anaemia or iron deficiency in the past 2 years? 81

Q9 - To your knowledge, have you ever had anaemia or iron deficiency in the past 2 years?	Percentage	Count
Yes	47%	38
No	53%	43

To your knowledge, have you ever had anaemia or iron deficiency in the past 2 years? 81

To your knowledge, have you ever had anaemia or iron deficiency in the past...	Average	Minimum	Maximum	Count
Yes	2.00	2.00	2.00	38
No	3.00	3.00	3.00	43

Have you taken oral iron tablets in the past 2 years? 81

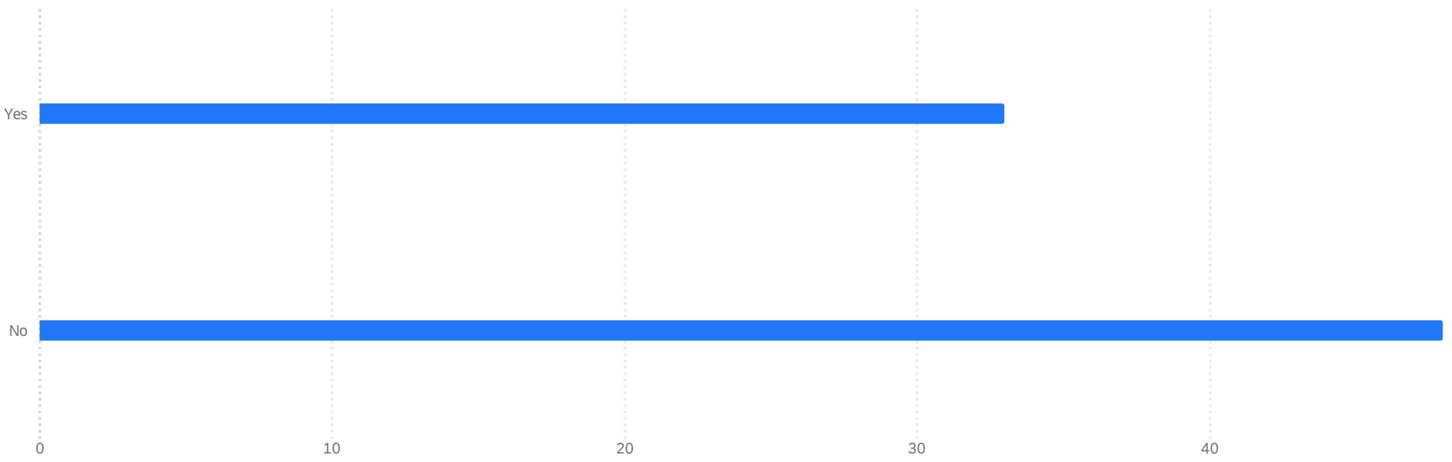

Have you taken oral iron tablets in the past 2 years? 81

Q10 - Have you taken oral iron tablets in the past 2 years?	Percentage	Count
Yes	41%	33
No	59%	48

Have you taken oral iron tablets in the past 2 years? 81

Have you taken oral iron tablets in the past 2 years?	Average	Minimum	Maximum	Count
Yes	1.00	1.00	1.00	33
No	2.00	2.00	2.00	48

Have you ever had an iron infusion? 81

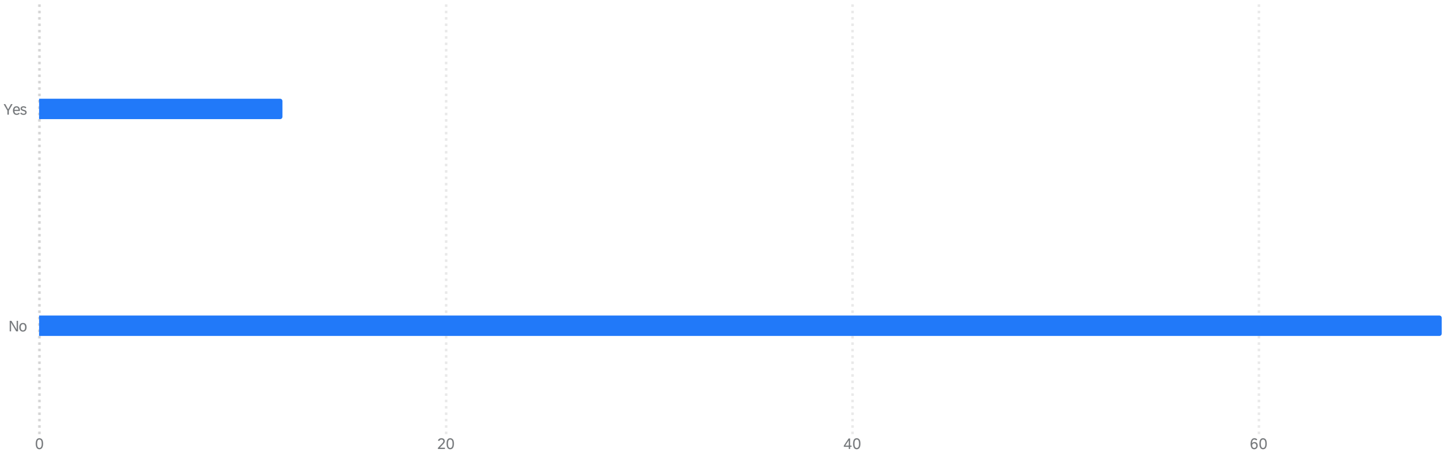

Have you ever had an iron infusion? 81

Q11 - Have you ever had an iron infusion?	Percentage	Count
Yes	15%	12
No	85%	69

Have you ever had an iron infusion? 81

Have you ever had an iron infusion?	Average	Minimum	Maximum	Count
Yes	1.00	1.00	1.00	12
No	2.00	2.00	2.00	69

How many years ago was your iron infusion? 81

How many years ago was your iron infusion?
N/A
N/A
N/A
N/A
N/A
N/A
1
N/A
N/A
N/A

How many years ago was your iron infusion?
N/A
N/A
N/A
N/A
N/A
N/A
N/A
6
N/A
N/A
N/A
N/A
N/A
N/A
N/A
N/A
N/A
N/A
N/A
N/A
N/A
N/A
N/A
N/A
N/A
N/A
N/A
N/A
N/A
1

How many years ago was your iron infusion?
N/A
0
N/A
N/A
N/A
N/A
4
N/A
N/A
2
N/A
3
N/A
N/A
N/A
N/A
N/A
N/A
N/A
N/A
N/A
N/A
N/A
N/A
N/A
1.5
N/A
N/A
N/A
N/A

How many years ago was your iron infusion?
N/A
1
N/A
5
N/A
N/A
1
N/A
1
N/A
N/A

Approximately how many periods have you had in the last 12 months? 56

Approximately how many periods have you had in the last 12 months?	Average	Minimum	Maximum	Count
Number of periods	8.68	0.00	16.00	56

In regards to your period, have you ever experienced any of the following? (Please select all that apply) 81

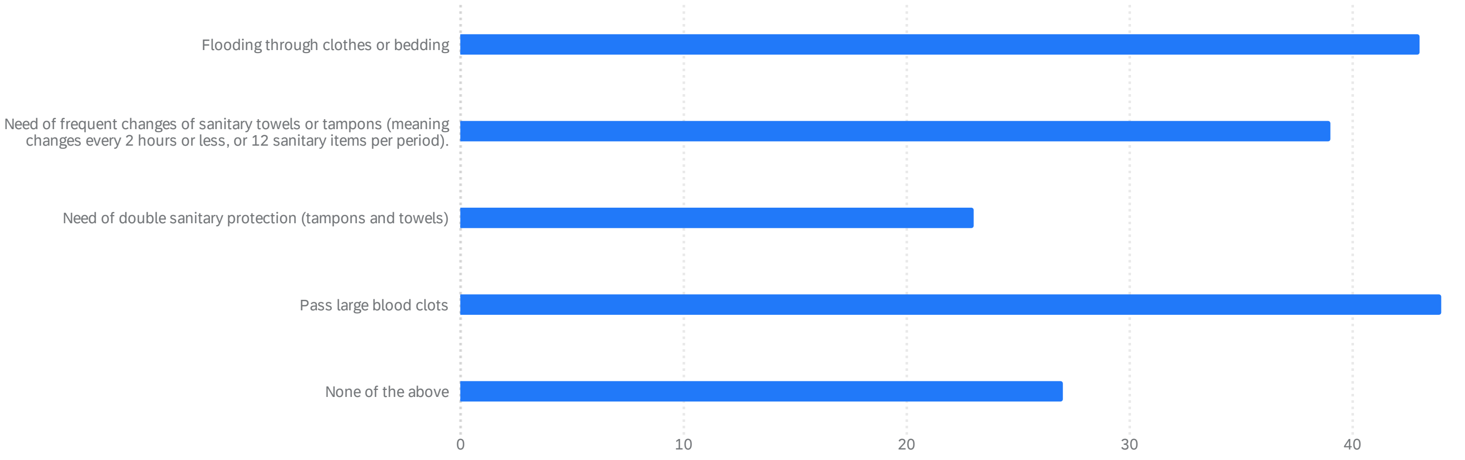

In regards to your period, have you ever experienced any of the following? (Please select all that apply) 81

Q14 - In regards to your period, have you ever experienced any of the following? (Please select all that apply)	Percentage	Count
Flooding through clothes or bedding	53%	43

Q14 - In regards to your period, have you ever experienced any of the following? (Please select all that apply)	Percentage	Count
Need of frequent changes of sanitary towels or tampons (meaning changes every 2 hours or less, or 12 sanitary items per period).	48%	39
Need of double sanitary protection (tampons and towels)	28%	23
Pass large blood clots	54%	44
None of the above	33%	27

Below are a list of symptoms- What are your symptoms of iron deficiency? (Please pick all that apply to you) 81

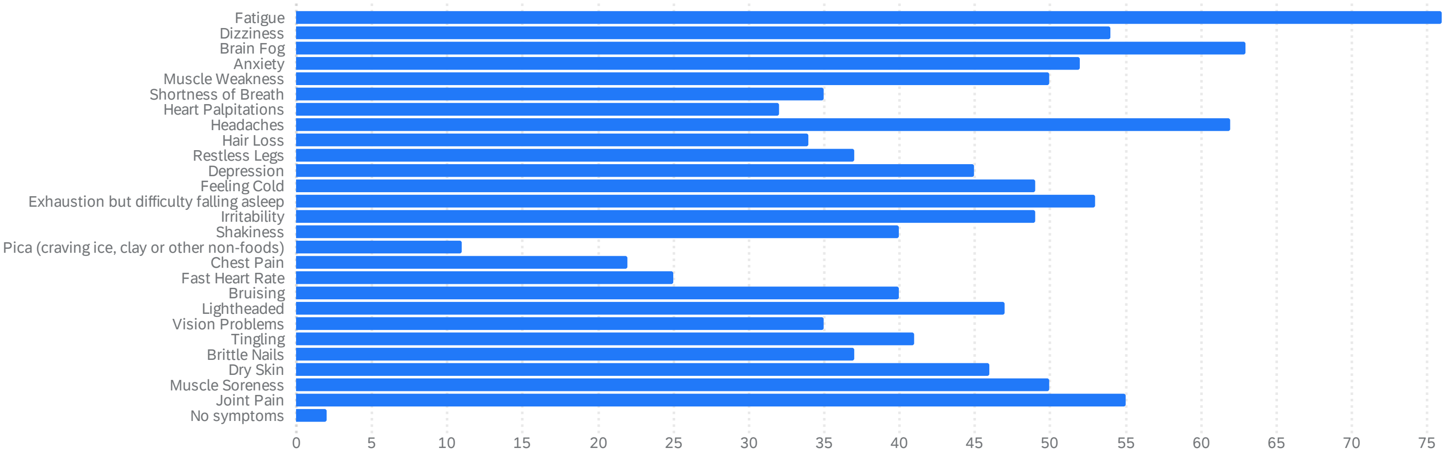

Below are a list of symptoms- What are your symptoms of iron deficiency? (Please pick all that apply to you) 81

Q15 - Below are a list of symptoms- What are your symptoms of iron deficiency? (Please pick all that apply to you)	Percentage	Count
Fatigue	94%	76
Dizziness	67%	54
Brain Fog	78%	63
Anxiety	64%	52
Muscle Weakness	62%	50
Shortness of Breath	43%	35
Heart Palpitations	40%	32
Headaches	77%	62
Hair Loss	42%	34
Restless Legs	46%	37
Depression	56%	45
Feeling Cold	60%	49

Q15 - Below are a list of symptoms- What are your symptoms of iron deficiency? (Please pick all that apply to you)	Percentage	Count
Exhaustion but difficulty falling asleep	65%	53
Irritability	60%	49
Shakiness	49%	40
Pica (craving ice, clay or other non-foods)	14%	11
Chest Pain	27%	22
Fast Heart Rate	31%	25
Bruising	49%	40
Lightheaded	58%	47
Vision Problems	43%	35
Tingling	51%	41
Brittle Nails	46%	37
Dry Skin	57%	46
Muscle Soreness	62%	50
Joint Pain	68%	55
No symptoms	2%	2

Have you donated blood in the past 2 years? 80

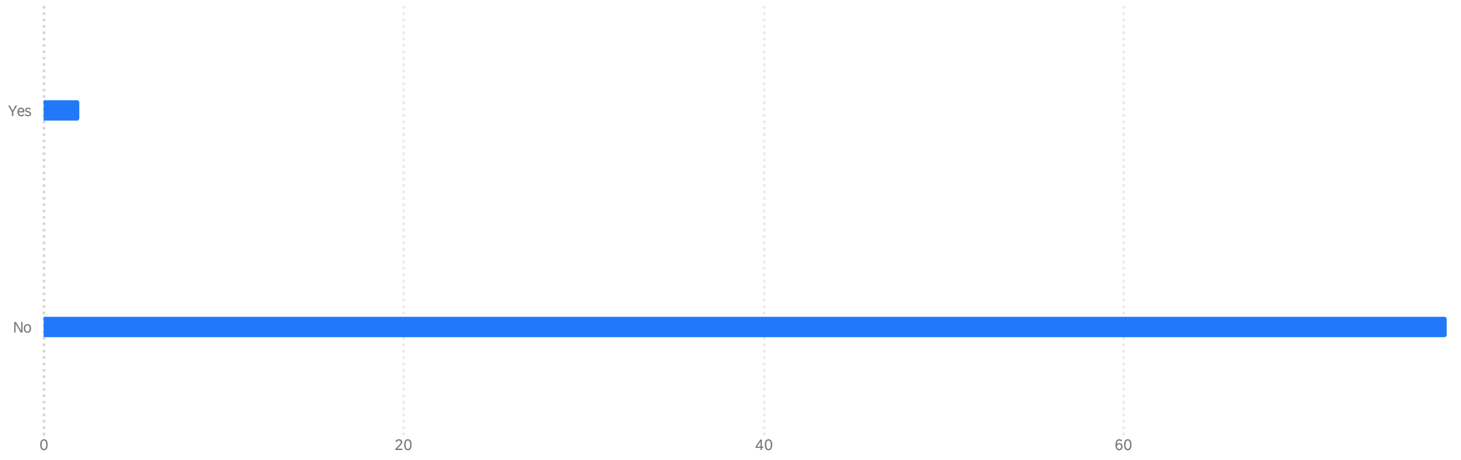

Have you donated blood in the past 2 years? 80

Q16 - Have you donated blood in the past 2 years?	Percentage	Count
Yes	3%	2
No	98%	78

Have you donated blood in the past 2 years? 80

Have you donated blood in the past 2 years?	Average	Minimum	Maximum	Count
Yes	1.00	1.00	1.00	2
No	2.00	2.00	2.00	78

Do you follow a vegetarian, vegan, pescatarian or similar diet? 81

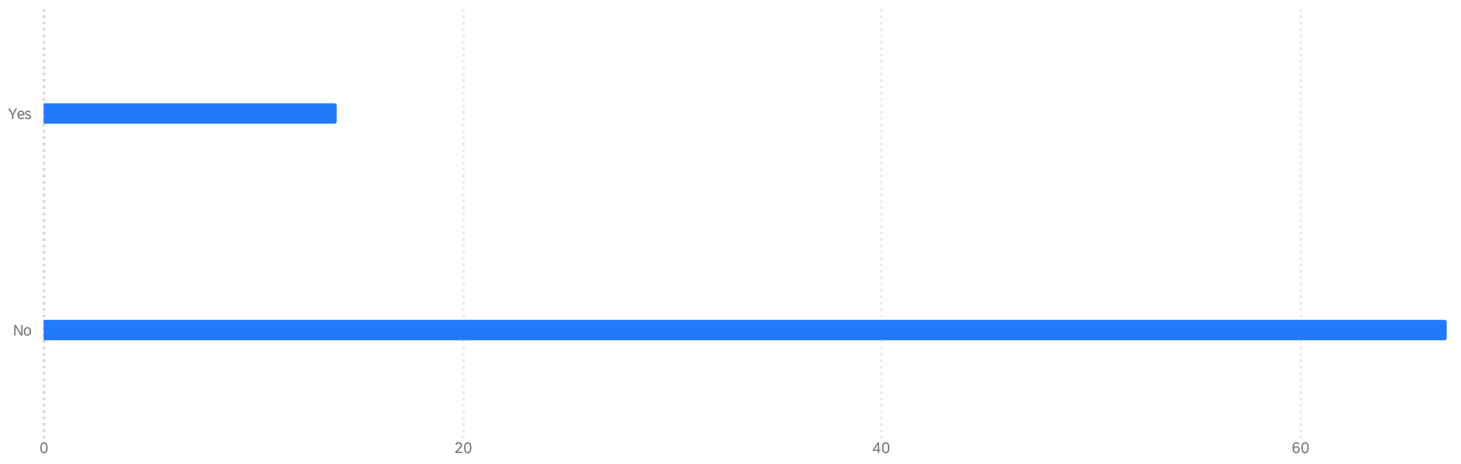

Do you follow a vegetarian, vegan, pescatarian or similar diet? 81

Q17 - Do you follow a vegetarian, vegan, pescatarian or similar diet?	Percentage	Count
Yes	17%	14
No	83%	67

Do you follow a vegetarian, vegan, pescatarian or similar diet? 81

Do you follow a vegetarian, vegan, pescatarian or similar diet?	Average	Minimum	Maximum	Count
Yes	1.00	1.00	1.00	14
No	2.00	2.00	2.00	67

Have you ever had a pregnancy? 81

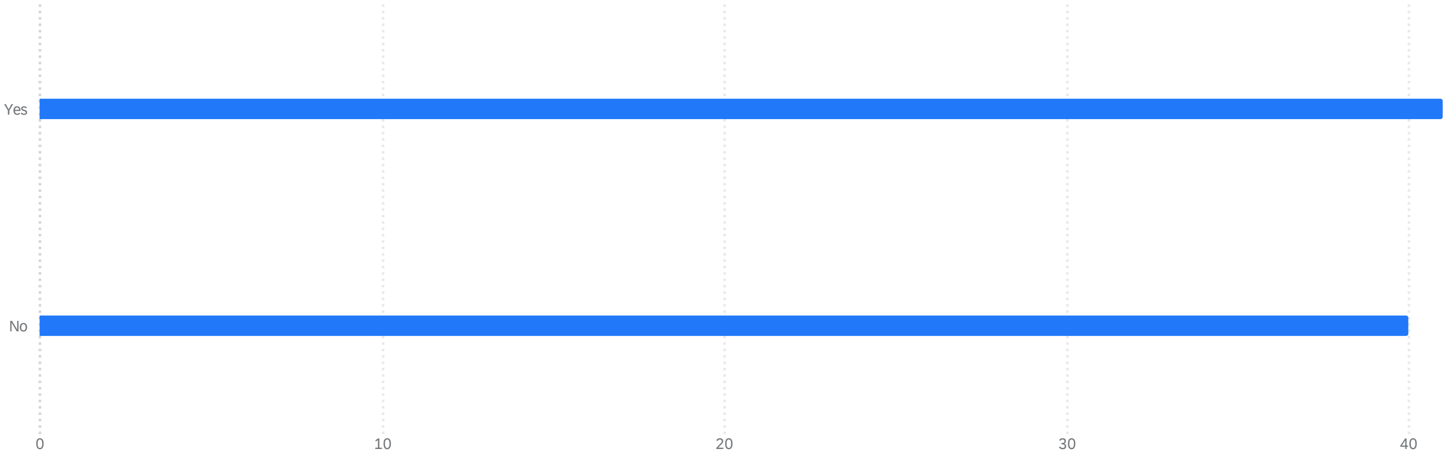

Have you ever had a pregnancy? 81

Q18 - Have you ever had a pregnancy?	Percentage	Count
Yes	51%	41
No	49%	40

Have you ever had a pregnancy? 81

Have you ever had a pregnancy?	Average	Minimum	Maximum	Count
Yes	1.00	1.00	1.00	41
No	2.00	2.00	2.00	40

How many children do you have? 41

How many children do you have?	Average	Minimum	Maximum	Count
Number of children	1.90	0.00	4.00	41

How many years ago was your last child born? 81

How many years ago was your last child born?
9
N/A
N/A
N/A
9
12
N/A

How many years ago was your last child born?
12
N/A
N/A
N/A
N/A
N/A
N/A
15
N/A
N/A
12
N/A
7
27
0
3
N/A
N/A
N/A
N/A
22
N/A
12
9
N/A
10
N/A
10
N/A
13

How many years ago was your last child born?
3
N/A
9
16
N/A
19
N/A
6
N/A
N/A
18
2
19
5
12
N/A
17
10
N/A
N/A
16
14
5
19
N/A
N/A
11
N/A
20
10

How many years ago was your last child born?
25
N/A
N/A
11
2
N/A
N/A
N/A
17
N/A
19
N/A
12
N/A

At your last childbirth, were you known to be iron deficient or anaemic? 41

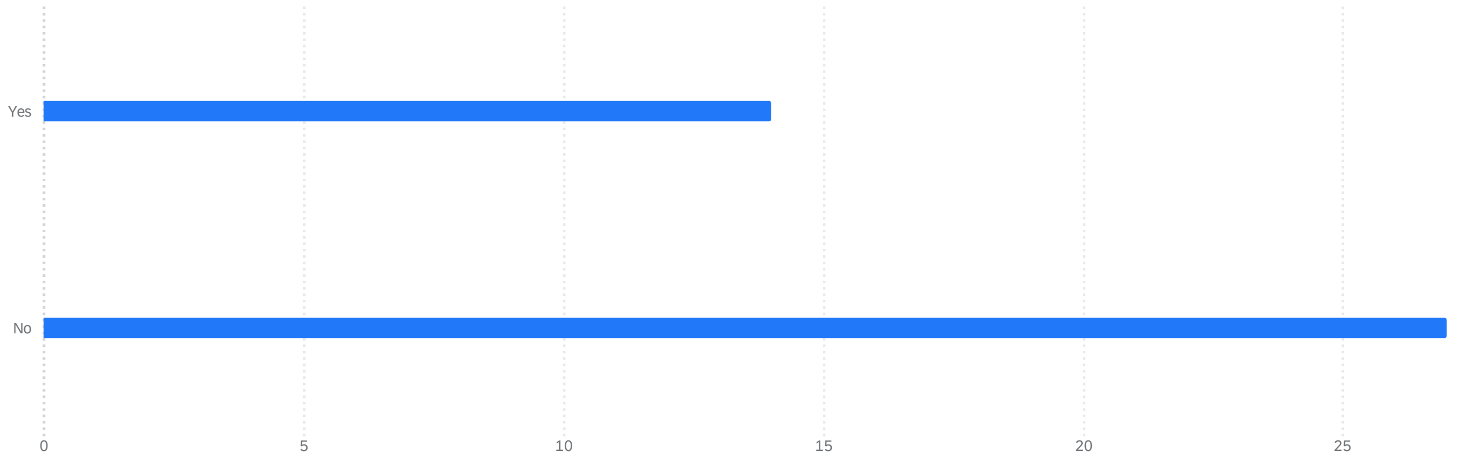

At your last childbirth, were you known to be iron deficient or anaemic? 41

Q21 - At your last childbirth, were you known to be iron deficient or anaemic?	Percentage	Count
Yes	34%	14
No	66%	27

At your last childbirth, were you known to be iron deficient or anaemic? 41

At your last childbirth, were you known to be iron deficient or anaemic?	Average	Minimum	Maximum	Count
Yes	1.00	1.00	1.00	14
No	2.00	2.00	2.00	27

At your last childbirth, did you suffer from hair loss? 41

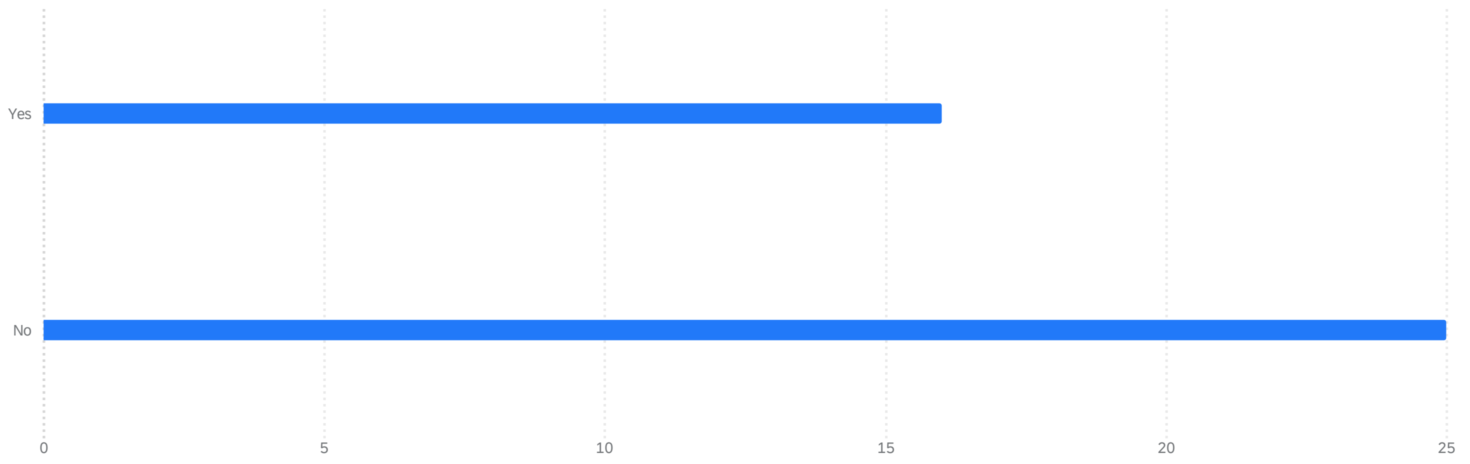

At your last childbirth, did you suffer from hair loss? 41

Q22 - At your last childbirth, did you suffer from hair loss?	Percentage	Count
Yes	39%	16
No	61%	25

At your last childbirth, did you suffer from hair loss? 41

At your last childbirth, did you suffer from hair loss?	Average	Minimum	Maximum	Count
Yes	1.00	1.00	1.00	16
No	2.00	2.00	2.00	25

At your last childbirth, did you suffer from postpartum depression? 41

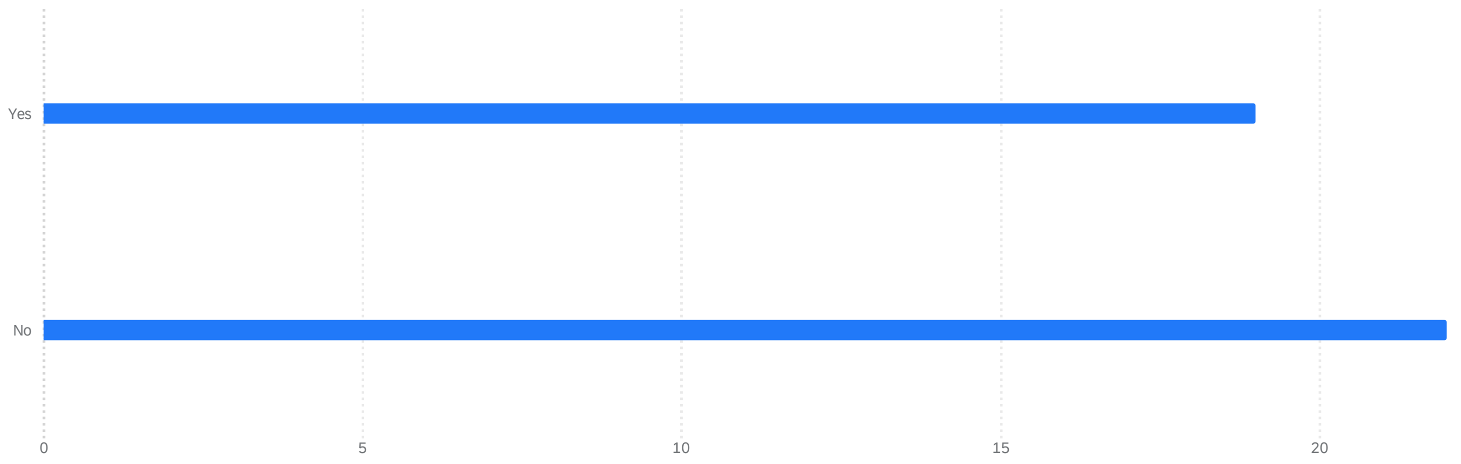

At your last childbirth, did you suffer from postpartum depression? 41

Q23 - At your last childbirth, did you suffer from postpartum depression?	Percentage	Count
Yes	46%	19
No	54%	22

At your last childbirth, did you suffer from postpartum depression? 41

At your last childbirth, did you suffer from postpartum depression?	Average	Minimum	Maximum	Count
Yes	1.00	1.00	1.00	19
No	2.00	2.00	2.00	22
